# The Psychometric Properties of Emotional Development Assessment tools in Intellectual Disabilities: A Systematic Review

**DOI:** 10.1111/jir.70023

**Published:** 2025-08-12

**Authors:** Bethany Leal, Mark Hudson

**Affiliations:** ^1^ College of Health and Science, School of Psychology, Sarah Swift building University of Lincoln Lincoln UK; ^2^ School of medicine, Mental Health and Clinical Neurosciences, Yang Fujia building Nottingham UK

**Keywords:** *assessment*, *challenging behaviour*, *emotional development*, *Intellectual disabilities*, *psychometric properties*, *systematic review*

## Abstract

**Background:**

People with intellectual disabilities can experience psychological distress and show behaviours of concern, such as self‐injurious behaviour or physical aggression. One contributing factor is the degree to which their emotional needs are understood by those in their environment. This paper aims to review the psychometric properties of assessment tools measuring emotional development in individuals with intellectual disabilities.

**Methods:**

A systematic literature review was conducted, which included 5 databases and followed the PRISMA guidance (registration number: CRD42024553322). Seven assessment tools were included in this review: the SAED, SED‐S, Brief SED‐S, SED‐R, and SED‐R^2^, SEO‐Lukas and the Frankish model, and the psychometric properties were assessed in accordance with the COSMIN good measurement properties checklist.

**Results:**

Sixteen studies were included in this review. Internal consistency was assessed in six of the seven measures; validity was only assessed in the SAED and SED‐S. Whilst both of these measures were considered reliable and valid, studies on the SAED had greater methodological quality, and the SED‐S had a larger quantity of evidence.

**Conclusions:**

Both the SAED and the SED‐S are psychometrically sound tools, based on the overall quality and sufficiency of the evidence. Further research should consider the usability, sensitivity and cross‐cultural use, especially in UK populations.

## Summary

- Most assessment tools in this review were considered reliable, except for the Frankish model.
- The SAED and SED‐S tools were the most reliable and valid based on quality, the amount of evidence, and thorough evaluation.
- Having a valid and reliable tool is important to ensure accurate assessments and provide the best support for people with intellectual disabilities.
- More studies should look at how these tools work in different cultures to make sure they can be used worldwide.

## 1 Introduction

### 1.1 Intellectual Disabilities and Behaviours of Concern

Understanding the emotional needs of individuals with intellectual disabilities plays an important role in helping to reduce the use of restrictive practices, by creating an opportunity to expand our awareness, and improving their quality of life (de Bruijn et al. 2021). There are approximately 1.3 million people with intellectual disabilities living in the UK (Office for Health Improvement & Disparities 2025), with the global prevalence rate at 1–2% (Maulik et al. 2011). Intellectual disability is defined by the American Association of Intellectual and Developmental Disabilities (AAIDD; 2021) as “a condition characterised by significant limitations in both intellectual functioning and adaptive behaviours that originates before the age of 22”. Individuals with intellectual disabilities are more likely to have comorbid mental health conditions, such as anxiety and depression (Mazza et al. 2020) as well as other neurodevelopmental conditions, such as Autism Spectrum Disorder (ASD; Baio et al. 2018) and Attention Deficit/Hyperactivity Disorder (ADHD; Ahuja et al. 2013). These conditions are likely to add greater burden on quality of life for individuals with intellectual disabilities, compared to the general population (Buckley et al. 2020). Research that aims to increase understanding of individuals with intellectual disabilities, their use of behaviours of concern, and their mental health often focuses on cognitive skills and adaptive behaviour (Alexander and Reynolds 2020). However, other contributing factors, such as their emotional needs, have historically received little attention.

Emotional needs can be expressed through behaviours of concern, particularly by those with limited communication (Hollo et al. 2014). Behaviours of concern, also known as challenging behaviour, can be defined as culturally abnormal behaviour of such intensity and frequency that physical safety is likely at risk (Emerson and Bromley 1995). Common examples of these behaviours are physical aggression, self‐injurious behaviour or property damage. There is a high association between intellectual disabilities and behaviours of concern, with the severity of the intellectual disability and additional disorders, such as autism spectrum disorders (ASD), being predictors for higher levels of behaviours of concern (Holden and Gitlesen 2006; Hill and Furniss 2006). (Murphy et al. 2005) Sappok et al. (2012) utilised a case‐study to demonstrate the impact of assessing and intervening around emotional needs. They concluded this helped others to understand the behaviours of concern and develop integrative treatment approaches for individuals with intellectual disabilities, ultimately providing a deeper understanding of the individual and improving their quality of life through their care and social relationships. Emotional needs have been linked with higher levels of behaviours of concern (Cole 2002), which highlights the significance of addressing this issue.

Compared to adult inpatients without intellectual disabilities, individuals in the United Kingdom (UK) with intellectual disabilities experience longer inpatient stays, higher rates of seclusion and segregation, and repeated admissions (Oxley et al. 2013), which is usually a result of the continued use of behaviours of concern. In response to the Care Quality Commission's (CQC) national review of hospitals for individuals with intellectual disabilities, the UK government set out to address failings in the care of individuals with intellectual disabilities and autism through the Transforming Care agenda. The Transforming Care report (Department of Health 2015) aimed to increase community provisions and care for people with intellectual disabilities in the UK, in recognition of the fact that hospital settings are inappropriate for this client group (Glasby et al. 2024). Despite these efforts, approximately 2000 individuals with intellectual disabilities in the UK remained in inpatient hospitals at the end of January 2020 (Parkin 2023). In other European countries, the service provision for those with intellectual disabilities varies, with countries like Spain and Greece now moving away from institutionalised care. However, the needs of the intellectual disability population are expected to be met in generic services (Spain) or services with no specialist multi‐disciplinary input (Greece) (Holt et al. 2000). Following the Transforming Care agenda, more work has been completed to understand individuals with intellectual disabilities and their needs through an attachment lens (Rinaldi et al. 2022). This has been deemed important to provide consistent care environments, which is more likely to reduce distress (National Institute for Health and Care Excellence (NICE) 2015). However, the use of restrictive practices such as physical restraint and seclusion are high in intellectual disability populations across almost all settings (Merineau‐Cote and Morin 2013), (Fitton and Jones 2020). According to the Learning Disabilities Census report, 47% of incidents (reported within 3 months of census) involved either physical restraint or seclusion (Health and Social Care Information Centre 2015). Although attempts to address these numbers have occurred over the last several years, these reports highlight the need for change in how individuals with intellectual disabilities are supported with their mental health needs.

Alternative care models are essential to address unmet needs and reduce restrictive practice in this population. Clegg and Lansdall‐Welfare (2023) highlighted the inadequacy of behavioural interventions to address behaviours of concern and emphasised the importance of recognising the emotional needs of those with intellectual disabilities, who require supportive and healthy relationships. While practical needs of individuals with intellectual disability are reported as “mostly met” emotional and relational needs are often not (Schützwohl et al. 2016). Whilst developmental approaches have been studied for several decades, their implementation has been slow in Anglophone countries. Clegg and Lansdall‐Welfare (2023) suggest that the historical inequality between the treatment of typically developing children versus those with intellectual disabilities has contributed to this.

### 1.2 Emotional Development

Emotional development (ED) can be defined as a child's developing capacity to manage relationships, express and experience their emotions in socially and culturally acceptable ways (Yates et al. 2008). Specific constructs such as emotional regulation (the ability to manage our own feelings; (Halle and Darling‐Churchill 2016) often form part of the wider ED domain. The stages of ED are well established when it comes to the typical development of children, and correlate with the child's chronological age, developing brain structures, and cognitive functioning (Malik and Marwaha 2018). This enables the child to engage in increasingly sophisticated relationships and develop skills such as object permanence and Theory of Mind (Mercer 2005). Emotional networks within the brain develop alongside social and cognitive networks, with each network impacting on one another's development (Pessoa 2010), although emotional experience can be considered a primary driver of subsequent mental abilities (Greenspan 1997). ED is important to consider when trying to understand an individual's behaviour in relation to others; for example, an individual who is yet to develop object permanence may struggle to develop trusting attachments with caregivers (Bell 1970).

Došen (2005) applied the concept of ED to people with ID through his dynamic‐developmental approach, which integrates ideas from numerous developmental theorists, including Bowlby, Ericksson, and Greenspan. The model outlines five developmental stages (see Table 1) based on typical children's development, which include: (1) adaptation, (2) socialisation, (3) individuation, (4) identification and (5) reality awareness (Došen 2005). A sixth stage, social individuation, was later added (Sappok et al. 2022). Frankish (1992) also outlined a psychodynamic approach to emotional development (also known as the Frankish model), which is based on developmental phases (see Table 1) and draws on the work of Mahler. The primary difference between the models is the range of domains that are covered, with Došen covering a wider range of developmental stages and Frankish only covering development up to three years (Table 1). Došen's model is also interested in the development of the brain (Došen 2018) and Frankish focusses mainly on the expression of trauma (McInnis 2016). Both models described in Table 1 consider the impact of ED on individuals with intellectual disabilities explicitly, and therefore are most suitable for this review.

**TABLE 1:** Stages of emotional development.

Stage (age)	As described by Došen (2005) and Sappok et al. (2022)	As described by Frankish (2019)
**1** **(0–6 months)**	*Adaptation:* Integration of sensory stimuli; integration of structures of time, place and persons; satisfies basic needs	*Differentiation*: Early developmental stage of self‐referenced behaviours; exploration of self and immediate surroundings; does not seek attention but responds to others
**2** **(7–18 months)**	*Socialisation:* Object permanence; secure attachment; development of body schemas	*Practising*: Starts to learn skills through repeating; Little reciprocal interaction; behaviour is still very self‐referenced, but shows signs of valuing others' attention.
**3** **(19–36 months)**	*Individuation:* Exploration from a secure base; autonomy; self‐other differentiation; discovering one's own will.	*Early rapprochement*: Meaningful two‐way interactions; takes note of impact of behaviour on others; actively seeks attention and begins to make choices; increased response to praise; development of autonomy without ability to reason. *Late rapprochement*: Negotiating; testing boundaries; start of delayed gratification; social interaction and reasoning develop rapidly; increased independence to point of rapprochement crisis.
**4** **(4–7 years)**	*Identification*: Theory of mind; interaction with peers; identifying with important others; differentiation between fantasy and reality	
**5** **(8–12 years)**	*Reality awareness*: Reflective thinking; logical reasoning; moral action; assessment of own abilities.	
**6** **(13–17 years)**	*Social individuation:* Identity‐formation; seeking role in society; internalised social rules	

Current research considers the legitimacy of assessing emotional development in individuals with intellectual disabilities. (Hermann et al. 2022) conducted research into the behavioural phenomena of individuals with intellectual disabilities and their emotional development level. They concluded that certain phenomena were specific to the level of emotional development, and concluded that this could be used to distinguish cause of the behavioural problem. Frankish (2013) used a behavioural observation model to identify developmental stage of the individual with intellectual disabilities, based on the Frankish model of emotional development. It was reported that once the stage had been identified, an appropriate “regime” could be put in place to support the individual to progress through developmental stages.

### 1.3 Emotional Development Assessment

Based on the above, ED assessment can be considered an important part of the overall assessment process for people with intellectual disabilities. However, current approaches to assessment in the UK focus on an individual's IQ and adaptive functioning, meaning that clinicians are missing vital information for understanding the strengths and needs of individuals with intellectual disabilities.

To date, there are limited tools that exist to assess the level of emotional development in individuals with intellectual disabilities. However, it is evident that emotional development is crucial to a person's overall functioning. Available tools include the Scale of Emotional Development – Short (SED‐S; Sappok et al. 2019) and the Frankish Assessment of the Impact of Trauma (FAIT; Frankish 2019). A review conducted by (Gourley and Yates 2022) identified both the SED‐S and FAIT as useful tools to support the psychological wellbeing of individuals with intellectual disabilities. They conclude that the FAIT was a more psychometrically supported measure, although the FAIT was only evaluated in a small number of studies. Future research recommended the psychometric properties of these tools be adequately investigated once additional research had been conducted.

### 1.4 Rationale and Aim of Review

Previously, (Gourley and Yates 2022) had conducted a literature review of the psychometric properties of therapeutic tools for individuals with intellectual disabilities, however, no systematic literature reviews have examined the psychometric properties of ED assessment tools for individuals with intellectual disabilities. This is important in guiding clinicians wishing to assess ED and for the implementation of the ED approach in Anglophone countries, where it is still in its infancy. The current literature base for emotional development assessment tools is small, however several tools have been developed. It is important that the quality of these tools is assessed systematically to ensure that clinicians can have confidence in their findings. This review aimed to appraise the psychometric properties of emotional development assessment tools used in intellectual disability populations, and assess the quality of this evidence.

## 2 Methods

### 2.1 Protocol and Registration

This systematic review was registered with PROSPERO (registration number: CRD42024553322) and the Preferred Reporting Items for Systematic Reviews and Meta‐Analyses 2020 (PRISMA) guidance was followed (Page et al. 2021; Appendix A).

### 2.2 Eligibility criteria

The eligibility criteria can be seen in Table 2. The research team had access to authors fluent in European Languages (Dutch and German) that could provide translation. Grey literature and non‐peer reviewed articles were excluded from this review as these sources can introduce uncorrected bias (Simundić 2013), however, doctoral theses were included in order to avoid publication bias.

**TABLE 2:** Inclusion and exclusion criteria for systematic searching of studies.

	Inclusion	Exclusion
**Population**	Intellectual disabilities or professionals working in intellectual disabilities, any age range	Non‐intellectual disabilities (mixed samples were included if ID sub‐group data could be extracted)
**Publication type**	Peer‐reviewed article; doctoral theses	Grey literature, non‐peer‐reviewed, abstracts
**Language**	European language papers	None
**Measurement tool**	Assessment of emotional development	None
**Design**	Quantitative or mixed methods	None
**Other**	Must report psychometric properties	None

### 2.3 Search Strategy

Six databases were systematically searched up to January 2025, including: PsychInfo, MEDLINE, CINHAL, Web of Science, Scopus and OpenDissertations. There were no limitations set on publication date. These databases were included as they cover a range of disciplinary areas, such as psychology, healthcare, medicine and social sciences, allowing the review to have a wider breadth.

The search terms (Table 3) were developed using PICO (Richardson et al. 1995) guidelines, scoping searches and key words from relevant papers investigating intellectual disabilities and emotional development. Guidance was sought from a specialist librarian to ensure search terms were relevant. The databases that used their own index terms (MEDLINE and CINHAL) were searched using the initial strategy plus additional index terms identified using the ‘AND’ operator (Table 3).

**TABLE 3:** Search strategy for systematic database searching.

Search terms	Terms combined using AND
Intellectual Disability	“intellectual disabil*” OR “learning disabil*” OR “mental retardation” OR “developmental disabil*” OR “intellectual developmental disorder” OR “developmental disabilities”
Additional index terms for MEDLINE	(MH “Intellectual Disability/PX”) OR (MH “Persons with Mental Disabilities/PX”) OR (MH “Learning Disabilities/PX”) OR (MH “Developmental Disabilities/PX”)
Additional index terms for CINHAL	(MH “Intellectual Disability/PF”) OR (MH “Persons with Intellectual Disabilities/PF”)
Emotional Development	“emotional development*” or “socioemotional functioning”
Additional index terms for MEDLINE	(MH “Emotional Intelligence”) OR (MH “Emotional Adjustment”)
Additional index terms for CINHAL	(MH “Emotional Intelligence”) OR (MH “Emotional Regulation”)

*Note:* Whilst the term “mental retardation” is outdated and has been superseded in the literature, this has been included in the search terms due to the open‐ended publication date range.

### 2.4 Study Selection

**FIGURE 1:**
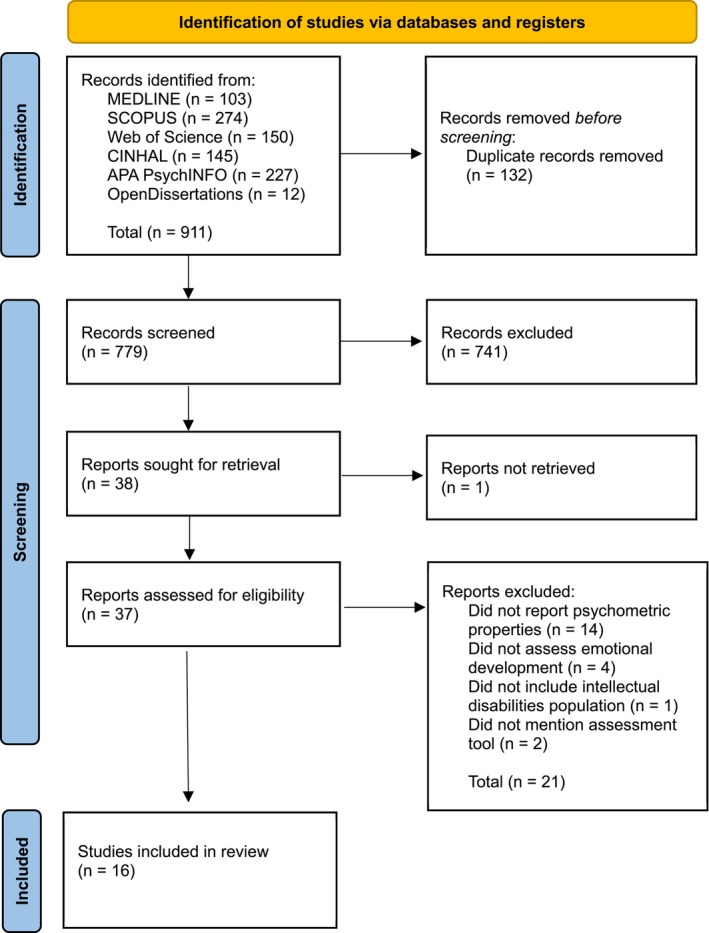
PRISMA diagram of systematic search.

Papers that were identified after systematic searching were exported into the online referencing software RefWorks (Proquest 2024), where they were de‐duplicated. The titles were screened against the eligibility criteria, followed by the abstracts and full texts. This process was completed by the first author. In cases where study suitability was unclear, a joint decision was made regarding inclusion or exclusion with the second author (see Figure 1 for the PRISMA diagram). Experts in the field were consulted to provide clarity on the existing literature and help in identifying any additional papers.

### 2.5 Data Extraction

Data was extracted by the first author into a data extraction form and included the following: Author, year, location, aim, assessment tool used, number of participants, age (mean and range), setting of population, methodology and psychometric findings. See Table 4 for results.

**TABLE 4:** Characteristics of included studies.

	Author, Year, Location	Aim(s)	Assessment tool	Participants	Methods	Findings
1	La Malfa et al. 2009 Italy	a) correlate the SAED with the VABS b) test the psychometric characteristics of the SAED	SAED	*N* = 33 adults (27% female, 73% male) *M a*ge = 39.5 Range = 20–59 Level of intellectual disabilities Moderate 46%, severe 51%, profound 3% Setting: Residential centres for intellectual disabilities	Emotional development and adaptive functioning assessed by trained expert clinicians. Internal consistency measured using Cronbach's Alpha Convergent validity of VABS and SAED measured using Person's correlation	**Internal consistency** Cronbach's alpha = 0.95 **Convergent validity** Pearson's correlation = *r =* 0.512, *p* = 0.002
2	Frankish 2013 UK	To establish the reliability and validity of the Frankish model assessment tool	The Frankish model	*N* = 13 adults (30% male, 70% female) *M* age = missing Range = 18–54 Setting: Secure and community	Observation and rating by pairs of graduate psychologists for 40 min Inter‐rater reliability measured with *K* statistic	**Inter‐rater reliability** Observed behaviours *K* = 0.47–0.88 (moderate to perfect) Stage of Emotional development = 100% perfect agreement (No *K* reported)
3	Sappok et al. 2013 Germany	Hypotheses: a) the overall level of ED in adults with intellectual disabilities/ASD combined is reduced compared to adults with intellectual disabilities alone. b) ASD is associated with specific deficits in certain domains of ED c) this pattern could be of value in predicting ASD group membership	SAED	*N =* 289 adults (35.6% female, 64.4% male) *M* age = 34.9 Range = not reported Level of intellectual disabilities Mild = 26.9% Moderate = 42.5% Severe‐profound = 30.45% Setting: In/out‐patient psychiatric hospital	SAED applied during routine care. Psychometric properties of the developed SAED algorithm were assessed by sensitivity, specificity, likelihood ratio of Cohen's kappa. The new algorithm for ASD group membership was measured using the receiver operating characteristic (ROC) and area under curve (AUC).	**Construct validity** Correlation with level of intellectual disabilities Spearman's rho = intellectual disabilities negatively correlated with ED (*rs* = ‐0.53, *p* < 0.001). Both severity of intellectual disabilities (*rs* = 0.28, *p* < 0.001) and level of ED (*rs* = ‐0.46, *p* < 0.001) correlated with ASD diagnosis
4	Elstner et al. 2016 Germany	a) Does the level of emotional and cognitive development differ? b) What is the internal consistency and the intra‐ and inter‐rater reliability of the team‐based approach?	SAED	*N* = 225 adults (53.7% male, 46.3% female) *M* age = 36.7 Range = not reported Setting: Specialised treatment centre (inpatient) intellectual disabilities and Psychiatric hospital across two wards	SAED applied as part of routine care. Assessed by full team rather than an individual. *Team‐based approach* Internal consistency measured using Cronbach's alpha. *Individual approach* Intra‐rater reliability = rated by one part of one rater pair, repeated by same person after one week. Inter‐rater reliability = both raters applied SAED within three days for same patient	**Internal consistency** Using the team‐based approach: Cronbach's alpha = 0.94 **Inter‐rater reliability** = *k*_*w*_ = 0.3–0.8 **Intra‐rater reliability** = *k*_*w*_ = 0.7 for over 7 dimensions
5	Sappok et al. 2016 Germany/Belgium/Netherlands	To develop a short, psychometrically sound instrument for assessing levels of ED in individuals with intellectual disabilities which can be applied to adults	SED‐S	*N* = 30 Expert professionals from the NEED group Inclusion: expert knowledge of developmental psychology and ED, extensive diagnostic experience with intellectual disabilities and experience administering SED‐R, SED‐R^2^, SAED	Online survey, consensus meetings and refinement stages Mean and Standard deviation of Likert‐scale (0 = high—3 = low) responses to validity and observability questions NEED group met for consensus meetings—multi‐site and cross‐cultural intelligibility	**Content Validity** Domains were assessed by *M* and *SD*. Only domain one, stage one reported as example: *M =* 1.35–1.77.
6	Vandevelde et al. 2016 Belgium	To describe the development of the SED‐R and assess its reliability	SED‐R	*N* = 67 children and adults (56% male, 34% female) *M* age = 34.3 Range = 7–75 Missing = 10 Level of intellectual disabilities Mild = 36% Moderate = 41% Severe = 17% Profound = 5% Missing = 9 Setting Residential care = 54% Home in community = 13% Foster care = 4% Supported living = 3% Missing = 23%	SED‐R carried out by two interviewers at different points within 1–3 weeks Internal consistency measured using Cronbach's alpha Test–retest reliability used Spearman's rho Inter‐rater reliability used intraclass correlation coefficients (weighted kappa coefficient)	**Internal consistency** Cronbach's alpha = 0.95 **Test–retest reliability** Spearman's Rho (between individual interviewers) = *r*(57) = 0.75 **Inter‐rater reliability** *k*_*w*_ = 0.76 for average score
7	Sappok et al. 2019 Germany	To assess the psychometric properties of the SED‐S	SED‐S	*N* = 160 children (50.7% female, 49.3% male) *M* age = 3.75 Range = 0–12 Settings: kindergartens, schools, sports clubs, day‐care and personal connection	SED‐S applied in semi‐structured interview from two psychologists. Criterion validity measured by weighted kappa. Internal consistency measured by Cronbach's alpha Inter‐rater reliability was assessed in 25 cases by two raters.	**Criterion validity** Domain level: *k*_*w*_ = 0.92–0.94. Overall classification: *k*_*w*_ = 0.95 **Internal consistency** Cronbach's alpha = 0.99 **Inter‐rater reliability** Two domains *k*_*w*_ = 0.98, others were *k*_*w =*_ 1
8	Sappok et al. 2020 Germany/Belgium/Netherlands/UK/Italy/Switzerland	To develop standardised index cases, one for each level of ED, to support the calibration of the assessors to improve the standardisation of the application of the scale on an international level	SED‐S	*N* = 23 Members of NEED group that developed index cases *N =* 20 Standardised index cases Familiarity with cases Familiar *n* = 11 Unfamiliar *n* = 9	23 NEED members developed index cases 5 video case descriptions rated by 20 NEED members across 6 countries. Written case summaries used. Those with SD > 0.5 were rephrased Inter‐rater reliability measured using ICC	**Inter‐rater reliability** ICC Domain: 0.85–0.94 ICC overall: 0.94 **Inter‐rater agreement** 81.8% across all cases and dimensions
9	Sterkenburg et al. 2021 Netherlands/Switzerland	Examine the reliability and validity of the SED‐S in children with intellectual disabilities	SED‐S	*N* = 118 children (66% male, 34% female) *M* age = 10.9 Range = 3–17 *Netherlands* 3 care organisations across east, west and south. *Switzerland* 3 care organisations Level of intellectual disabilities Mild = 25% Moderate = 39% Severe = 27% Profound = 8% Unknown = 1% Setting: Living with parents = 64% Group homes = 23% Care facilities = 11% Unknown = 1%	Semi‐structured interview with someone who knew the child well Internal consistency measured using Cronbach's Alpha Validity was examined using Goodman and Kruskal's *γ*, Kruskal‐Wallis *H*, and Mann–Whitney *U* tests	**Internal consistency** Cronbach's alpha = 0.94 **Reliability** Domain correlation: γ = 0.63–0.86. **Convergent validity** SED‐S with severity of intellectual disabilities: *G* = −0.69, *p* < 0.001. **Divergent validity** SED‐S with chronological age = (*H*[4] = 9.25, *p* = 0.055) SED‐S with adaptive age = (*ƿ*[46] = 0.614, *p* < 0.001)
10	Hermann et al. 2022 Germany	To specify the behavioural phenomena that are typical for the level of ED in a larger sample of 185 adults with intellectual disabilities	SED‐S	*N* = 185 adults (male 63.2%, female, 36.8%) *M* age = 36.5 Range = 18–65 Level of intellectual disabilities Mild = missing Moderate = 31.9% Severe = 42.2% Profound = missing Setting: Inpatient and outpatient clinic	Retrospective study where SED‐S was used routinely Cronbach's alpha was used to measure internal consistency	**Internal consistency** Cronbach's alpha = 0.92
11	Tarasova et al. 2022 Germany	To define the SED‐S items for stage 6	SED‐S	*N* = 28 Expert members of NEED group	56 items were developed across 3 languages by members of NEED group Online survey completed to assess content validity and observability The ratings were analysed by calculating means (*M*), standard deviations (*SD*), medians, and minimum and maximum scores. Items with the lowest mean content validity and observability scores were eliminated	**Content validity** Stage 1–5 *M* = 0.1–1.45 Stage 6 *M* = 0.06–0.61
12	Flachsmeyer et al. 2023 Belgium/Netherlands/Germany	To assess the factor structure and internal consistency in adults with intellectual disabilities To determine its reliability and the construct validity in a multicentred design	SED‐S	*N* = 724 adults (male 56%, female 44%) *M* age = 37.4 Range = 18–76 Level of intellectual disabilities Mild = 28.2% Moderate = 37.4% Severe = 26.8% Profound = 7.6% Setting: Recruited from hospital and sheltered living institutions	Level of ED was assessed using the SED‐S by trained clinicians in an interview. Construct validity: Confirmatory factor analysis was used, and a one‐factor model was tested. Fit was evaluated using chi‐squared. Invariance was measured across sex, ASD group membership and level of intellectual disabilities. One‐factor model was considered to hold if chi‐squared was non‐sig. and CFI was < 0.001 Internal consistency measured using Cronbach's alpha	**Construct validity** One‐factor model fitted data well, *X* ^*2*^ = 32.12, df = 20, *p* = 0.019, CFI = 0.999 **Invariance measurement** Good model fit (Configural, metric and scalar) across all groups except profound ID. **Internal consistency** Cronbach's alpha = 0.93
13	Sappok et al. 2023 Germany/Belgium	To compare the level of emotional functioning as assessed using the SED‐S, with the SAED, SED‐R2 and SEO‐Lukas	SED‐S, SAED, SED‐R^2^ SEO‐Lukas	*Berlin* *n* = 85 (male 62.4%, female 37.6) *M* age = missing Range = missing Level of intellectual disabilities Mild = 10.6% Mod = 42.4% Severe = 36.5% Profound = 4.7% *Liebenau* *n* = 51 (male 51%, female 49%) *M* age = 37 Range = missing Level of intellectual disabilities Mild = 33.3% Mod = 45.1% Severe = 15.7% Profound = 5.9% *Belgium* *n* = 50 (male 18%, female 82%) *M* age = 44.4 Range = missing Level of intellectual disabilities Mild = 34% Mod = 44% Severe = 22% Profound = 0%	Emotional development was measured in adults with the SED‐S (*n* = 186), SAED (*n* = 85), SED‐R^2^ (n = 50) and SEO‐Lukas (n = 51). Internal consistency was measured used Cronbach's alpha. Convergent validity of the SED‐S was compared against the SAED, SEO‐Lukas and SED‐R^2^ using weighted kappa	*SAED* **Internal consistency** Cronbach's alpha = 0.91 *SEO‐Lukas* **Internal consistency** Cronbach's alpha = 0.97 *SED‐R* ^*2*^ **Internal consistency** Cronbach's alpha = 0.95 *SED‐S* **Internal** **Consistency** Cronbach's alpha = 0.93 **Convergent validity** With SAED *k*_*w*_ *=* 0.34 With SEO‐Lukas *k*_*w*_ = 0.93 With SED‐R^2^ *k*_*w*_ = 0.18
14	Sappok et al. 2024 Germany	To develop a brief version of the SED‐S	Brief SED‐S	*N* = 447 adults *M* age = 36.9 Range = 18–76 Settings: Patients from the Berlin Center for Mental Health in Developmental Disabilities and the St. Lukas‐Klinik, Liebenau	Two samples developed, sample one to find the most appropriate items, sample two to validate. Internal consistency: Kuder–Richardson formula used due to the dichotomy of the items. Validity: relative and exact agreements per level of ED, the overall agreement and weighted kappa, and sensitivity, specificity and accuracy were calculated.	**Internal consistency** Kuder–Richardson Cronbach's alpha = 0.3–0.8 for item analysis, *M* = 0.88 **Overall agreement** *k*_*w*_ = 0.743, *P*_*O*_ = 0.7 **Sensitivity** *M* = 0.681 **Specificity** *M* = 0.918 **Accuracy** *M* = 0.88
15	Hermann et al. 2024 Germany/Netherlands	To explore the psychometric properties of the items on the SED‐S by answering: What is the frequency of ‘yes’ answers to each item on the scale, what are the sensitivity and specificity rates of each item on the scale, and what is the discriminatory power of each item on the scale?	SED‐S	*N* = 612 adults (59.8% male, 40.2% female) *M* age = 37.4 Range = 18–72 Level of Intellectual Disabilities: Mild: 27% Moderate: 35.6% Severe: 28.6% Profound: 8.8% Settings: 2 hospitals, 4 care homes, residential facilities and day care centres.	SED‐S used by trained individuals (mostly psychologists) with at least two informants. The specificity, sensitivity and discriminatory power of each item on the scale was measured by examining the frequency of yes answers by individuals in respective domain stage, no answers outside of respective domain stage and comparison of yes answers in respective stage versus adjacent stages.	**Content validity** **Sensitivity** for all 200 items average = 67.3% (*SD* = 17.16, range 19–97) **Specificity** for all 200 items on average = 79.3% (*SD* = 13.21, range 37–99) **Discriminatory power:** 90% of items discriminated significantly against adjacent domains
16	Meinecke et al. 2024 Germany	To analyse and standardise the SED‐S in adults with ID without comorbid mental disorders in non‐clinical sample	SED‐S	*N* = 83 adults (60.2% male, 39.8% female) *M* age = 39.1 Range = 19–76 Level of Intellectual Disabilities: Mild: 20.5% Moderate: 28.9% Severe: 20.5% Profound: 30.1% Settings: psychiatric hospital/sheltered workplace	Measures applied by a clinical psychologist; SED‐S with one or two informants; DAS with informants; Spearman's rank and Kruskal‐Wallis used for associations Confirmatory factor analysis used for construct validity Cronbach's alpha used for internal consistency	**Associations** *p* < 0.001 (*Rs* = −0.753) 62.65% expected SED‐S stage for level of ID **Construct validity** Chi‐squared goodness of fit non‐significant (*X* ^*2*^ = 8.388, df = 20, *p* = 0.989), all eight domains significantly predicted by one factor estimate ranging from 0.750 to 0.934, one factor model was upheld **Internal consistency,** Cronbach's alpha = 0.92 (CI 0.89, 0.94)

*Note:* Definitions of abbreviations: SAED = Scheme for Appraisal of Emotional Development; SED‐S = Scale of Emotional Development—Short; SED‐R = Scale of Emotional Development—Revised; SED‐R^2^ = Scale of Emotional Development—Revised, Second Edition; SEO‐Lukas = Schaal voor Emotionele Ontwikkeling—Lukas; ED = Emotional Development; ASD = Autism Spectrum Disorder; VABS = Vineland Adaptive Behaviour Scale; NEED = Network of Europeans for Emotional Development; ICC = Intraclass correlation coefficient, CFI = Comparative fit index.

### 2.6 Quality Appraisal

The methodological quality of the studies was assessed using the COSMIN Risk of Bias checklist (Mokkink et al. 2018; available at www.cosmin.nl). This tool was chosen as it was developed exclusively for use in systematic reviews of measurement properties. The tool enables the reviewer to determine whether studies can be trusted, based on their methodological quality. The reviewer categorises the methodological quality of the studies into four categories, first appraising the study per measurement property and then giving an overall category based on the worst score. Each study has been rated and coloured as either “very good” (dark green□), “adequate” (light green◊), “doubtful” (orange○) or “inadequate” (red●). The first author completed the risk of bias assessment which was then second‐rated by the second author, any differing ratings were discussed until a conclusion had been reached.

### 2.7 Psychometric property assessment

The psychometric properties of each study were extracted and then evaluated individually against the COSMIN criteria for good measurement properties checklist (Mokkink et al. 2010; Appendix B). Based on this, each psychometric property per study was given a rating and colour of “+” (green; sufficient result), “?” (orange; indeterminate result) or “‐“(red; insufficient result). Each psychometric property has its own criteria, for example, sufficient results for internal consistency are reported to be a Cronbach's α of ≥ .7 and “at least low quality evidence for sufficient unidimensionality”, an indeterminate result is “Criteria not met for at least low quality evidence of unidimensionality” and an insufficient result is a Cronbach's α < 0.7 and at least low evidence for sufficient structural validity. The COSMIN guidance uses a taxonomy of measurement properties (see Appendix C), indicating which measures hold the most weight for providing good evidence of psychometric properties. Additional COSMIN criteria (Terwee et al. 2018; available at www.cosmin.nl) were used to consider content validity and measure development.

Content validity is considered the most important psychometric measure, followed by the internal structure of the measure (internal consistency and measurement invariance; see Appendix D for definition), and then the rest of the measurement properties in the taxonomy.

### 2.8 Grading of Evidence

The final stage of assessment summarised the evidence for each assessment tool and used the GRADE approach (Guyatt et al. 2008). The overall summary of findings (SoF) per measurement property was scored against the guidance for good measurement properties (Mokkink et al. 2010) and rated as sufficient “+”, insufficient “‐“, inconsistent “±” or indeterminate “?”. Then, the quality of the evidence per measurement property was graded against 4 categories: risk of bias, inconsistency, imprecision, and indirectness. This provided the quality rating for the overall evidence of specific measurement properties per assessment tool. High quality evidence suggests that further research is unlikely to change the confidence in the estimated effect, and low quality suggests that any effect estimate is uncertain (Guyatt et al. 2008; see Appendix E).

### 2.9 Data Analysis

The COSMIN guidance for systematic reviews of Patient‐Reported Outcome Measures (PROMs; Prinsen et al. 2018) was used as a framework for the structure of this systematic review. The type of instrument to be reviewed was not included in the search strategy, as this may have limited results. Due to the heterogeneity and small number of the studies included in this review, a narrative synthesis was used to analyse and report results. Following the guidance set out by Popay et al. (2006), the initial theory pertaining to the importance of psychometric assessment in the area was developed, before developing a preliminary synthesis of the findings in the subsequent tables. The relationships between measurement instrument, psychometric properties and methodological qualities were explored and robustness was measured. The aim was to include all nine psychometric properties, as recommended by the COSMIN guidance. However, available results precluded a full review and seven of the recommendations were therefore included.

## 3 Results

### 3.1 Search Results and Study Characteristics

899 studies were identified before eligibility criteria were applied. The full search process is detailed in the PRISMA diagram (Figure 1). Sixteen studies using seven emotional development measures were included in this review (Table 4). Six measures were based on the Došen (2005) theory of emotional development (SAED; SEO‐Lukas; SED‐R; SED‐R^2^; SED‐S, Brief SED‐S), whilst one measure used the Frankish (2013) model. Table 5 provides a brief explanation of the seven assessment tools. Three studies focused on the measure development with an expert group, while 13 studies reviewed the measure within the target population. Of the 13 studies, 11 recruited from adult populations and two used a child population. The sample size ranged from small (*n* = 13; study 2) to large (*n* = 724; study 12).

**TABLE 5:** Brief description of Emotional Development measures.

Measure Name	Brief description
**Frankish model**	Observational model of assessment; four stages of ED; used to develop targeted intervention based on stage of ED identified
**Scheme for Appraisal of Emotional Development (SAED)**	Observation or informant interview; ten domains of psychosocial development across five stages of ED; scored using yes/no answers; used to identify potential cause of behavioural problems
**Schaal voor Emotionele Ontwikkeling – Lukas (SEO‐Lukas)**	Observational and semi‐structured interview; six stages of ED across eight domains; scored using yes/no answers; used to identify ED profile and target intervention
**Scale of Emotional Development – Revised (SED‐R)**	Adaptation of the SAED for better accessibility for daily use with practitioners; 13 domains across five stages of ED; scored using behavioural observation and informant discussion
**Scale of Emotional Development – Revised. Second edition (SED‐R** ^**2**^ **)**	Second revision of the SED‐R to improve applicability, accuracy and validity; eight domains across five stages of ED; scored using behavioural observation and informant discussion
**Scale of Emotional Development – Short (SED‐S)**	Condensed version of the SED‐R^2^ to allow for assessment in time‐limited settings; semi‐structured, questionnaire‐based interview; scored using yes/no answers; eight domains across five stages of ED
**Brief Scale of Emotional Development—Short**	Intended to be used as an initial screening tool, gives an overall score rather than domain specific; can be applied by other professions; brief, quicker application than others.

All studies were conducted in Europe. Some studies were single‐site (Germany, studies 3, 4, 7, 10, 11, 14, 16; United Kingdom [UK], study 2; Italy, study 1; Belgium, study 6) and the others were multi‐national (Germany and Belgium, study 13; Germany and The Netherlands, study 15; Switzerland and The Netherlands, study 9; three or more nations, studies 5, 8, 12).

### 3.2 Quality Appraisal

Using the COSMIN Risk of Bias checklist (Mokkink et al. 2018), seven studies were rated overall as very good, one study was adequate, four studies were doubtful, and four studies were inadequate. The overall rating is taken as the lowest quality psychometric property rating for that study. Table 6 shows the full results per measurement property for quality appraisal.

**TABLE 6:** Quality appraisal of studies using COSMIN Risk of Bias checklist.

Study no.	Assessment tool	PROM development	Content validity	Internal consistency	Measurement invariance	Reliability	Criterion validity	Construct validity	Overall quality rating
**1**	SAED			Very good○				Very good□	Very good□
**2**	Frankish	Inadequate●				Doubtful○			Inadequate●
**3**	SAED							Very good□	Very good□
**4**	SAED			Very good□		Very good□			Very good□
**5**	SED‐S	Doubtful○	Adequate◊						Doubtful○
**6**	SED‐R	Doubtful○		Very good□		Doubtful○			Doubtful○
**7**	SED‐S			Very good□		Adequate◊	Very good□	Very good□	Adequate◊
**8**	SED‐S					Very good□			Very good□
**9**	SED‐S			Very good□				Very good□	Very good□
**10**	SED‐S			Doubtful○					Doubtful○
**11**	SED‐S	Inadequate●	Doubtful○						Inadequate●
**12**	SED‐S			Very good□	Inadequate●			Very good□	Inadequate●
**13**	SED‐S, SAED, SED‐R^2^ SEO‐Lukas			Very good□				Very good□	Very good□
**14**	Brief SED‐S	Doubtful○		Very good□			Very good□		Doubtful○
**15**	SED‐S	Inadequate●	Doubtful○						Inadequate●
**16**	SED‐S			Very good□				Very good□	Very good□

### 3.3 Appraisal of Psychometric Properties

Of the eight measurement properties listed in the COSMIN guidance for good measurement properties (Mokkink et al. 2010), five were reported across the 16 studies included in this review: internal consistency, reliability (test–retest, inter/intra‐rater), criterion validity, construct validity and measurement invariance. Content validity and measure development were considered using additional COSMIN criteria (Terwee et al. 2018; publicly available at www.cosmin.nl). The measurement development (referred to as PROM development) is first considered for the relevant studies (*n* = 6) and used to determine overall rating for content validity.

#### 3.3.1 Measure development

Six studies covering four assessment tools (The Frankish model, SED‐S, SED‐R and the brief SED‐S) considered the development of the measure. The methodological quality was low, with ratings ranging from doubtful to inadequate. The SED‐S went through several stages of development, with changes being made including extending (study 11) and shortening (study 14). The extending study was rated as inadequate and the shortening was rated doubtful. The Frankish model development was rated as inadequate due to limited reporting of the methods in the study.

#### 3.3.2 Content validity

Content validity was considered in three studies reporting on the SED‐S (studies 5, 11 and 15). Studies 11 and 15 were rated as indeterminate, due to the overall methodology being inadequate. The content validity methodology was rated doubtful due to lack of interview or pilot tests results in the studies. Study 5 had doubtful overall methodological quality, and also showed indeterminate results for content validity.

#### 3.3.3 Internal consistency

The 10 studies that reported on the internal consistency all yielded sufficient results (Cronbach's α ≥ 0.7), across six out of the seven assessment measures (Table 7). The quality of the SAED was very good. The SED‐S reported the internal consistency as high across all six studies, however the quality of these studies was mixed (inadequate to very good). The remaining assessment tools (SED‐R, SED‐R^2^, Brief SED‐S, and SEO‐Lukas) were assessed in one study each and were rated as doubtful to very good.

**TABLE 7:** Internal consistency.

Assessment tool	Study no.	Psychometric Rating	Result (Cronbach's α =)	Overall Methodological quality
**SED‐S**	7	+	0.99	Adequate◊
	9	+	0.94	Very good□
	10	+	0.92	Doubtful○
	12	+	0.93	Inadequate●
	13	+	0.92	Very good□
	16	+	0.92	Very good□
**Brief SED‐S**	14	+	0.88	Doubtful○
**SAED**	1	+	0.96	Very good□
	4	+	0.94	Very good□
	13	+	0.90	Very good□
**SED‐R**	6	+	0.95	Doubtful○
**SED‐R** ^**2**^	13	+	0.95	Very good□
**SEO‐Lukas**	13	+	0.97	Very good□

*Note:* α = alpha.

#### 3.3.4 Reliability (test–retest or inter/intra‐rater reliability)

Five studies reported reliability, with results in question for the Frankish model and SED‐R, largely due to poor methodological quality. The SED‐S and SAED had higher methodological quality (Table 8), however, psychometric results for the SAED were indeterminate, while the SED‐S was sufficient. The Frankish model was of inadequate methodological quality, as well as having an indeterminate result. This rating was given because they did not report reliability of the measure in full or using the recommended statistic (Mokkink et al. 2010).

**TABLE 8:** Reliability (test–retest or inter/intra‐rater reliability).

Assessment tool	Study	Psychometric rating	Result		Overall Methodological quality
			Test–retest	Inter/intra‐reliability	
**Frankish model**	2	?		For observed behaviours κ = 0.47–0.88 For emotional development level 100% agreement	Inadequate●
**SAED**	4	?		Weighted κ = 0.3–0.8	Very good□
**SED‐R**	6	+	Spearman's Rho = 0.75	Weighted κ = 0.76	Doubtful○
**SED‐S**	7	+		Weighted κ = 1 (Exception of two domains at Weighted κ = 0.96)	Adequate◊
	8	+		Overall ICC = 0.942 Agreement = 81.8%	Very good□

*Note:* κ = kappa; ICC = intraclass correlation coefficient.

#### 3.3.5 Criterion validity

The criterion validity was measured by two studies. Studies assessing the SED‐S and Brief SED‐S did not report criterion validity using the recommended statistic (Mokkink et al. 2010). The quality of the SED‐S and Brief SED‐S evidence was varied (Table 9).

**TABLE 9:** Criterion validity.

Assessment tool	Study		Psychometric Rating	Result	Overall Methodological quality
**SED‐S**	7		?	Kappa was 0.95 for the overall classification, with an exact agreement of 80.6%	Adequate◊
**Brief SED‐S**	14		?	Mean sensitivity/specificity values were 0.681 and 0.918, respectively. The overall accuracy of the brief version of the SED‐S was 0.880	Doubtful○

*Note:* AUC = Area under curve.

#### 3.3.6 Construct Validity

The construct validity was assessed in seven studies, five reviewing the SED‐S and two reviewing the SAED. In studies 1 and 13, convergent validity was reported. Under the COSMIN guidance (Mokkink et al. 2010), this is considered part of construct validity measurement. The quality of the studies was inadequate to very good, and the results were all sufficient in their findings (Table 10).

**TABLE 10:** Construct validity.

Assessment tool	Study	Psychometric Rating	Result	Overall Methodological quality
**SAED**	1	+	Confirmed correlations between emotional development and adaptive abilities	Very good□
	3	+	Lower and uneven profile than intellectual disabilities alone. Can be used for ASD group membership	Very good□
**SED‐S**	7	+	Response probability within the target age group was sig. Higher compared to adjacent age groups	Adequate◊
	16	+	Eight domains of SED‐S defined as one construct and sig. Associated with level of ID	Very good□
	9	+	High internal consistency, inter‐dimensional correlation and correlation between level of ED and intellectual disabilities	Very good□
	12	+	Confirmatory factor analysis for domains estimates all significant	Inadequate●
	13	+	Matched overall score as assessed with long version of SED‐S	Very good□

*Note:* Sig. = Significantly.

#### 3.3.7 Measurement invariance

One study reviewing the SED‐S (study 12) reported on measurement invariance. The methodological quality of this study was rated as inadequate and the result was rated sufficient in measurement invariance across all groups (sex, ASD group membership, level of intellectual disabilities) except for profound level of intellectual disabilities, where the sample size of the group was too small to complete analysis.

### 3.4 Summarising and Grading the Evidence

Evidence was first evaluated per measurement property, per tool by being pooled or summarised together. The SoF per measurement property and quality of the evidence was then rated, providing a quality rating for overall evidence of measurement properties per tool. See results in Table 11.

**TABLE 11:** Overall measurement property sufficiency and evidence quality.

	Internal consistency		Reliability		Criterion validity		Construct validity		Measurement invariance	
	SoF	GRADE	SoF	GRADE	SoF	GRADE	SoF	GRADE	SoF	GRADE
**SAED**	+	HIGH	±	HIGH			+	HIGH		
**SED‐S**	+	HIGH	+	LOW	+	MOD	+	MOD	+	HIGH
**Brief SED‐S**	+	LOW			±	LOW				
**SED‐R**	+	MOD	+	MOD						
**SED‐R** ^**2**^	+	MOD								
**SEO‐Lukas**	+	MOD								
**Frankish**			−	VERY LOW						

*Note:* SoF = Summary of findings; SAED = Scheme for Appraisal of emotional development; SED‐S = Scale of emotional development – Short; SED‐R = Scale of emotional development—Revised; SED‐R^2^ = Scale of emotional development—Revised (Second edition); SEO‐Lukas = Schaal voor Emotionele Ontwikkeling—Lukas.

#### 3.4.1 SAED

The SAED was identified as having high quality evidence of reliability and validity, using the GRADE approach. The sample size varied across studies, but this did not have any effect on the internal consistency of the measure, which was significantly above the standard required. The inter/intra‐rater reliability of the measure was assessed in one identified study. The results were indeterminate due to methodological issues preventing an objective rating in at least one domain. The construct validity was also high quality and high standard.

#### 3.4.2 SED‐S, Brief SED‐S

The SED‐S was the most widely evaluated tool in this review, with 11 identified studies investigating the reliability and/or validity. The internal consistency was sufficient; however, the methodological quality of these studies mean that the overall reliability of this tool was moderate. The SED‐S has been identified as a reliable and valid tool by this review, whereas the literature for the Brief SED‐S was limited and could not give a comprehensive overview.

#### 3.4.3 SED‐R, SED‐R^2^

The SED‐R and SED‐R^2^ have been grouped together in this part of the analysis as one is the revised version of the other, with minimal changes between them. Both measures were only reviewed in one study each, with moderate quality. However, the results for reliability are consistent across these studies. The sample size for both studies were smaller in comparison to other measures, which means the true effect could have been different.

#### 3.4.4 SEO‐Lukas

Only one study was identified that measured the internal consistency of the SEO‐Lukas, which found this to be significantly above the required standard. The sample size for this study was smaller than other sub‐groups and it was unclear if the effect size was accounted for in the results.

#### 3.4.5 Frankish Model

The one study that reported psychometric properties of the Frankish model was very low quality. Whilst the agreement was reported to be 100%, the kappa statistic did not appear to be weighted and so the measurement property was rated as insufficient for reliability. The study claimed to assess validity but did not report any statistical information to evidence this. Based on this information the Frankish model was concluded to not be a reliable or valid measurement tool.

## 4 Discussion

This paper aimed to systematically review the psychometric properties of emotional development assessment tools within the intellectual disability population. It also aimed to review the quality of the evidence and grade this accordingly. Sixteen studies were included in this review. The most reviewed assessment tool was the SED‐S, with 11 studies including this. All tools had a form of reliability assessed (internal consistency, test–retest or inter/intra‐rater), but only the SED‐S and SAED was tested for validity (content/criterion/construct). The psychometric properties were assessed against the COSMIN criteria for good measurement properties (Prinsen et al. 2018). The methodological quality of the studies was measured using the COSMIN Risk of Bias checklist (Mokkink et al. 2018), using a “worst score counts” approach.

According to the COSMIN criteria, content validity is the most crucial psychometric property for a measurement tool (Terwee et al. 2018). Only four studies assessed content validity on a single measure (SED‐S), all of which were adequate or lower methodological quality. Similar to previous studies of psychometric properties (Osmancevic et al. 2021), these studies did not report asking about the comprehensiveness of the tool explicitly and were consequently rated doubtful or inadequate. Three studies used expert groups and multiple rounds of surveys to establish comprehensiveness and suitability, however the evidence was insufficient for a higher rating. The psychometric property rating of all four studies was indeterminate due to inadequate PROM development and lack of evidence of relevance, comprehensiveness, and comprehensibility. The recommendations include a qualitative investigation via a cognitive interview or pilot study, as part of the PROM development (Terwee et al. 2018). Given the limited number of studies and quality of the evidence, further, more robust, research is required to accurately assess the content validity of ED measures.

The overall quality of evidence for each tool ranged from high to very low across the six measurement properties. It is important to consider the timeline of scale development. Five of the assessment tools (SED‐R, SED‐R^2^, SEO‐Lukas, SED‐S and Brief SED‐S) are all revisions of the first scale developed, the SAED (Sappok et al. 2023). The quality of evidence of internal consistency for the SAED was high, based on the GRADE approach and the measurement property showed an overall sufficient result. The evidence for the following five tools showed high to moderate quality with the exception of the Brief SED‐S, who showed a low quality evidence. This could have been impacted by a very low (one) number of studies evaluating this tool. Internal consistency measures the correlation between items on the scale that aim to measure the same thing (Tavakol and Dennick 2011). For this to be assessed accurately, the construct being measured must be clearly defined within the literature (Kimberlin and Winterstein 2008). As the measurement of emotional development uses domain‐based ratings, each domain must be clearly defined. This has been a strength of the literature for all the studies included in this review that measure internal consistency and suggests the revisions of the first scale, SAED, have maintained this aspect well.

Only one study was identified in this review that measured the Frankish model (subsequently re‐named the Frankish Assessment of the Impact of Trauma or FAIT; Frankish 2019). The methodological quality of this study was rated very low, and the psychometric reliability was insufficient. The theoretical underpinning of this model is similar to that of Došen's model, but is less defined in the literature. After scoping searches, only three studies were found that reported using or reviewing this model of assessment (McInnis 2016; Gourley and Yates 2022; Frankish 2013) and only one was suitable for inclusion in this review. Not only did the study have an inadequate sample size (*n* = 13), the paper also left out important methodological and statistical information. Based on the results, the FAIT cannot be recommended for the assessment of emotional development.

### 4.1 Key strengths and Limitations

One strength of this research was the openness to including studies in multiple European languages and consultation with experts in the field. This allowed for a more robust search of the literature and a more accurate representation. Authors of identified research were consulted via email regarding the current literature and provided support in identifying any additional studies, although no additional studies were identified. Whilst doctoral theses were included, grey literature and non‐peer reviewed articles were not considered as part of this review. It should be acknowledged that one tool that was identified in addition to the tools included in this review was the Socio‐Emotional Developmental Age Level (SEDAL; Hoekman et al. 2015), which is the English translation of the Experimentele Schaal voor de beoordeling van het Sociaal Emotionele Ontwikkelingsniveau (ESSEON; Hoekman et al. 2007). However, the evaluations of psychometric properties for this tool are published in masters theses and therefore did not meet the inclusion criteria for this review. In regards to the methodology of this study, it is important to note that the guidance followed for assessing measure development was based on PROMs, whereas the tools assessed were not outcome measures. However, at the time of publishing this was deemed the most appropriate guidance to follow. One of the limitations of this review is the potential confirmation bias. Anton Došen described his theory of emotional development in several papers and books (e.g., Fletcher and Došen 1993; Došen and Day 2001; Došen 2005) and a group of researchers from across Europe were established to pursue interest in this (Network of Europeans on Emotional Development [NEED]). Most of the research included in this review has been conducted by the same authors, with several being part of this expert group. While some research suggests that this is not a limitation (Johnson 2018), it is important to consider the effects of confirmation bias during evaluation. Whilst the quality appraisal was conducted by both authors, the data extraction and analysis was conducted by only one reviewer. Though this review followed the strict protocol of the PRISMA (Page et al. 2021) and COSMIN (Prinsen et al. 2018) guidance, it still could be subject to reviewer bias.

### 4.2 Clinical Implications and Future Research

Given the range of tools available to measure emotional development, this systematic review has clinical relevance in enabling clinicians to identify which tool is the most reliable, based on the evidence. Whilst both the SAED and the SED‐S assessed reliability and validity, the SAED is currently the most robust measure for in‐depth evaluation of ED. However the SED‐S has sufficient psychometric properties to recommend its use, as a more time‐efficient measure in routine practice. Whilst understanding the psychometric properties of assessments tools is important, other factors should also be considered before using measures in clinical practice. Some of the studies in this review aimed to evaluate the tools in different populations (intellectual disabilities with no co‐morbidities, Meinecke et al. 2024; intellectual disability and ASD, Sappok et al. 2013) and some considered the sensitivity to change (Brief SED‐S, Sappok et al. 2024; SED‐S, Hermann et al. 2024), but a more detailed analysis should be considered. Future research should focus synthesising the data that is currently available about the usability and cultural applicability of these tools, and individual studies should focus on using the tools across different sub‐population groups. Since many tools have been used across different countries in Europe, future research must now focus on establishing the cross‐cultural validity in English‐speaking countries. Further research may focus on improving the methodological quality for future studies, by adhering to the recommended guidance (COSMIN; Mokkink et al. 2018) for study design and reporting psychometrics. They may also consider transparently addressing the development of the measure in future studies, by providing detailed accounts of piloting, testing and reviewing with population samples.

## 5 Conclusions

This systematic review assessed the psychometric quality of assessment tools for emotional development amongst an intellectual disability population. It concluded that both the SAED and the SED‐S are the most psychometrically sound tools, based on the overall quality and sufficiency of the evidence. This is an important finding for clinicians who regularly assess emotional development, or clinicians looking to implement this approach in their practice. This research further adds to the literature on the scientific assessment of emotional development in adults with intellectual disabilities.

## Conflicts of Interest

The authors declare no conflicts of interest.

## Supporting information


**Appendix S1:** Supporting Information

## Data Availability

The data that support the findings of this study are available from the corresponding author upon reasonable request.
